# 

**Published:** 1920-12

**Authors:** 


### The Library of the Bristol Medico-Chirurgical Society.

**The Library is now open from 9 a.m. to 7 p.m. except on Saturday, when it closes at 1 p.m.**

*December* 1*st*, 1920.

*The following donations have been received since the publication of the list in September.*

| | |
|---|---|
| The American Laryngological, Rhinological and Otological Society (1) .. .. .. | 1 volume. |
| Dr. J. R. Kay-Mouat (2) .. .. .. | 4 volumes. |
| Dr. Arthur B. Prowse (3) .. .. .. | 28 ,, |
| Dr. J. E. Shaw (4) .. .. .. .. | 1 volume. |
| Dr. J. O. Symes (5) .. .. .. .. | 1 ,, |
| Mr. J. Taylor (6) .. .. .. .. | 100 volumes. |
| J. Wright & Sons (7) .. .. .. .. | 1 volume. |

Unbound periodicals and reports have been received from Mr. Hey Groves, Mr. L. M. Griffiths, and the Medical Research Council.

### THE ONE HUNDRED AND SECOND LIST OF BOOKS.

The figures in brackets refer to the figures after the names of the donors, and show by whom the volumes were presented. The books to which no such figures are attached have either been bought from the Library Fund or received through the *Journal*.

| | | | |
|---|---|---|---|
| **Bardswell, N.** .. | *Handbook for Tuberculosis Workers* .. .. .. | | 1920 |
| **Bateman, T.** .. | *Delineations of Cutaneous Disease* .. .. | (3) | 1828 |
| **Beatty, J.** .. .. | *Method of Enzyme Action* .. .. .. .. .. | (2) | 1917 |
| **Bowlby, A. L.** .. | *Elementary Statistics* .. .. .. .. .. .. | (2) | 1910 |
| **Bradby, M. K.** .. | *The Logic of the Unconscious Mind* .. .. .. | | 1920 |
| **Bradley, S. M.** .. | *Manual of Comparative Anatomy* (6) | 3rd Ed. | 1875 |
| **Buckmaster and H. R. B. Hickman, G. A.** | *Course of Practical Physiology* .. .. .. .. .. .. .. .. | | 1920 |

**Carter, A. H.** .. *Practical Medicine* (Ed. by A. G. Gibson) 11th Ed. 1920
**Cope, Z.** .. .. *Surgical Aspects of Dysentery* .. .. .. .. 1920
**Cowper, W.** .. *The Anatomy of Humane Bodies* .. .. (5) 1698
**Cramer, W.** .. *Practical Chemical Physiology* .. .. 4th Ed. 1920
**Cretin, J.** .. .. *Lectures on Hygiene* .. .. .. .. .. .. (3) 1911
**Fincham, H. W.** *The Order of the Hospital of St. John of Jerusalem* .. .. .. .. .. .. .. (6) [1915]
**Fisher, T.** .. .. *The Heart and Sudden Death* .. .. .. (6) 1908
**Fortescue-Brickdale, J. M.** *Pharmacology and Medical Treatment for Nurses* .. .. .. .. .. .. .. 1920
**Fothergill, J. M.** .. *Diseases of the Heart* (6) .. .. .. 2nd Ed. 1879
„ .. *Chronic Bronchitis* .. .. .. .. .. .. (6) 1882
**Gallavardin, L.** .. *La Tension artérielle en clinique* (7) 2me Éd. 1920
**Garrod, A. B.** .. *Materia Medica* (3) .. .. .. .. 8th Ed. 1880
**Groves, E. W. H.** *Synopsis of Surgery* .. .. .. .. 5th Ed. 1920
**Hickman, G. A. Buckmaster and H. R. B.** *Course of Practical Physiology* .. .. .. .. .. .. .. 1920
**von Hoen, Ritter** .. *Strategical and Tactical Employment of the Medical Service* (Trans. by W. G. Macpherson) (2) 1909
**Hurst, A. F.** .. .. *Psychology of the Special Senses* .. .. .. .. 1920
**Ibbotson, W.** .. *Atlas of the Sensory Cutaneous Nerves* .. .. [1920]
**Jones, H. L.** .. *Medical Electricity* (Ed. by L. W. Bathurst) 6th Ed. 1920
**Lane, J. R.** .. .. *Lectures on Syphilis* (6) .. .. .. 2nd Ed. 1881
**Laumonier, J.** .. *New Methods of Treatment* (Trans. and Ed. by H. W. Syers) .. .. .. .. .. .. .. (6) 1904
**McCrae, W. Osler and T.** *Principles and Practice of Medicine* (4) 9th Ed 1920
**McNamara, C.** .. *Asiatic Cholera* .. .. .. .. .. .. .. (3) 1870
**Mann, M.** .. .. *Text-book of Tracheo-Bronchoscopy* (Trans. by A. R. Moodie) .. .. .. .. .. .. .. .. 1920
**Matthew, C. H. L. Rixon and D.** *Anxiety Hysteria* .. .. .. .. 1920
**Osler and T. McCrae, W.** *Principles and Practice of Medicine* (4) 9th Ed. 1920
**Pereira, J.** .. .. *Selecta è Prescriptis* .. .. .. (3) 14th Ed. 1864
*Pharmacopœia of Queen's Hospital for Children* .. .. .. 6th Ed. 1920
**Poore, G. V.** .. .. *Electricity in Medicine and Surgery* .. .. (6) 1876
**Rixon and D. Matthew, C. H. L.** *Anxiety Hysteria* .. .. .. .. 1920
**Ross, J.** .. .. *Aphasia* .. .. .. .. .. .. .. .. (3) 1887
**Souttar and E. W. Twining, H. S.** *Injuries of the Peripheral Nerves* 1920
**Spencer, H. R.** .. *Tumours Complicating Pregnancy etc.* .. .. 1920
**Sydenham, T.** .. *Works* (Corrected by J. Pechy) (6) .. 11th Ed. 1740
**Tibbles, W.** .. .. *The Theory of Ions* .. .. .. .. .. .. (2) 1908
**Twining, H. S. Souttar and E. W.** *Injuries to the Peripheral Nerves* 1920
**Wilson, E.** .. .. *Diseases of the Skin* (3) .. .. .. 4th Ed. 1857
**Wood, J.** .. .. *On Rupture* .. .. .. .. .. .. .. .. (6) 1863

TRANSACTIONS, REPORTS, JOURNALS, &c.

| | | |
|---|---|---|
| American Laryngological, Rhinological and Otological Society, Transactions of the | (1) | 1919 |
| American Surgical Association, Transactions of the | Vol. XXXVII. | 1919 |
| Johns Hopkins Hospital Reports, The | Vol. XIX. | 1920 |
| Journal of Physical Therapeutics (6) | Vols. II., III. | 1901–02 |
| Medical Electrology and Radiology (6) | Vols. IV.—VI. | 1903–05 |
| Smithsonian Report for 1917 | | 1919 |

### MEETINGS OF SOCIETIES.

### Bristol Medico-Chirurgical Society.

### Local Medical Note.

### Chesterfield Nursing Home and St. Brenda's Private Hospital.

With a view to extending the present Nursing Home to something near fifty beds the Trustees of the Bristol Nurses' Institute have acquired the entire block. We congratulate Dr. R. G. P. Lansdown, the Chairman, and Mr. Hedley Hill, the Hon. Treasurer, on carrying through this project, the success of which is mainly due to their skilful management and untiring effort.

We offer very hearty wishes for the success of St. Brenda's Private Hospital, which opens on January 24th. The Medical, Surgical and Special Staff form a complete team, each specialising in his own department of practice, who undertake the treatment of the relatively poor at very moderate fixed rates for maintenance and medical attendance. The two houses contain about forty beds, together with an operating theatre, X-ray and pathological installations. Mr. S. V. Stock, F.R.C.S., is at present acting as Registrar, and will furnish every information desired.

Both of these institutions aim at affording as far as possible the advantages of a modern hospital.

### INDEX.

Adsorption phenomena, The clinical significance of—Dr. O. C. M. Davis, 205.
Albuminuria, Non-nephritic, 162.
Amalgamation of Bristol hospitals, Proposed, 73, 130.
Appendicitis, Causation of, 241.
Arthritis, Traumatic, Treatment of, 55.

Bernard, D. E., Obituary notice of, 184.

Birrell, Dr. J. A.—Post-influenzal tachycardia, 122.

**Books, Reviews of**—

Alcoholism, Forms of—Dr. H. Wingfield, 62.

Allen, Dr. R. W.—Practical vaccine treatment, 67.

Anatomy, Surgical applied—Sir F. Treves, 66.

Back injuries—Dr. A. McKendrick, 64.

Bacteriology, Manual of—Drs. R. Muir and J. Ritchie, 128.

Basu, M. D.—Diabetes and its dietetic treatment, 177.

Bayliss, W. M.—Introduction to general physiology, 69.

Berkeley, Dr. C.—Diseases of women, 172; (with V. Bonney)—Guide to gynæcology in general practice, 72.

Bonney, Drs. C. Berkeley and V.—Gynæcology in general practice, 72.

Bipp treatment of war wounds—R. Morison, 65.

Borradaile, L. A.—Elementary zoology, 68.

Bradby, M. K.—Psycho-analysis and its place in life, 175.

Brown, Dr. W. Langdon—The sympathetic nervous system in disease, 228.

Burns and their treatment—Dr. J. M. H. MacLeod, 65.

Cataract, the Indian operation for—Dr. R. H. Elliot, 66.

Child welfare—Dr. J. S. Wallace, 224.

Clarke, Dr. J. M.—Neurological and other papers, 170.

Daw, S. W.—Orthopædic effects of gun-shot wounds, 225.

Dawson, Lord—The nation's welfare, 174.

Deane, H. E.—Gymnastic treatment for deformities, 65.

Diabetes and its dietetic treatment—M. D. Basu, 177.

Duval, P.—War wounds of the lung, 70.

Ear in school children, Diseases of the—Dr. J. K. Love, 225.

**Books, Reviews of** (*continued*)—
Elliot, Dr. R. H.—The Indian operation for cataract, 66.
Encyclopædia Medica, 71.
Eugenics and environment—C. L. Morgan, 127.
Fevers in the tropics—Sir L. Rogers, 59.
Field Service Handbook, Medical—C. M. Page, 65.
Findlay, Dr. L.—Syphilis in childhood, 177.
First aid, The soldiers'—R. C. Wood, 177.
Groves, E. W. H.—Surgical operations, 71.
Gymnastic treatment for deformities—H. E. Deane, 65.
Gynæcology in general practice—Drs. C. Berkeley and V. Bonney, 72.
Haldane, Dr. J. S.—The new physiology, 227.
Health, National—Dr. F. Rees, 66.
Indian operations for cataract—Dr. R. H. Elliot, 66.
Intestinal stasis, Operative treatment of chronic—Sir W. A. Lane, 63.
Joint deformities, Gymnastic treatment for—H. E. Deane, 65.
Journal of industrial hygiene, 174.
Lane, Sir W. A.—The operative treatment of chronic intestinal stasis, 63.
Lamb, Dr. W.—Diseases of the throat, nose and ear, 224.
Lettsom, J. C.—Sir St. C. Thomson, 173.
Love, Dr. J. K.—Diseases of the ear in school children, 225.
Lung, War wounds of the—P. Duval, 70.
McKendrick, A.—Back injuries, 64.
MacLeod, Dr. J. M. H.—Burns and their treatment, 65.
McVail, Dr. J. C.—Smallpox and vaccination, 176.
Military surgery—Dr. D. P. Penhallow, 63.
Morgan, C. L.—Eugenics and environment, 127.
Morison, R.—Bipp treatment of war wounds, 65.
Mosquitoe distribution in England, Map showing, 226.
Muir and J. Ritchie, Drs. R.—Manual of bacteriology, 128.
Mummery, J. H.—The microscopic anatomy of the teeth, 172.
Nation's welfare, The—Lord Dawson, 174.
Nauheim treatment, The—Dr. L. T. Thorne, 61.
Naval hospital ship, Administration of—E. Sutton, 129.
Nervous heart, The—Dr. R. M. Wilson, 129.
New physiology, The—Dr. J. S. Haldane, 227.

**Books, Reviews of** (*continued*)—
Orthopædic effects of gunshot wounds—S. W. Daw, 225.
Page, C. M.—A medical field service handbook, 65.
Paterson, Dr. A. M.—The anatomy of the peripheral nerves, 171.
Pear, Dr. G. E. Smith and T. H.—Shell-shock and its lessons, 226.
Penhallow, Dr. D. P.—Military surgery, 63.
Pensions, War diseases and—Drs. R. M. and W. M. T. Wilson, 176.
Peripheral nerves, Anatomy of—Dr. A. M. Paterson, 171.
Physiology, Introduction to general—W. M. Bayliss, 69.
Porter, Dr. W. G.—Diseases of the throat, nose, and ear, 68.
Power, Sir D'A.—Cancer of the tongue, 64.
Prognosis, Index of—Dr. A. R. Short, 127.
Psycho-analysis—M. K. Bradby, 175.
Pye's surgical handicraft, 62.
Rees, Dr. F.—National health, 66.
Ritchie, Drs. R. Muir and J.—Manual of bacteriology, 128.
Riviere, Dr. C.—The early diagnosis of tubercle, 61.
Rogers, Sir L.—Fevers in the tropics, 59.
Shell-shock and its lessons—Dr. G. E. Smith and T. H. Pear, 226.
Short, Dr. A. R.—Index of prognosis, 127.
Smallpox and vaccination—Dr. J. C. McVail, 176.
Smith and T. H. Pear, Dr. G. E.—Shell-shock and its lessons, 226.
Surgical handicraft, Pye's, 62.
Surgical operations—E. W. H. Groves, 71.
Sutton, E.—Administration of a naval hospital ship, 129.
Sympathetic nervous system in disease, The—Dr. W. Langdon Brown, 228.
Syphilis in childhood—Dr. L. Findlay, 177.
Teeth, Microscopic anatomy of the—J. H. Mummery, 172.
Thomson, Sir St. C.—J. C. Lettsom, 173.
Thorne, Dr. L. T.—The "Nauheim" treatment, 61.
Throat, nose and ear, Diseases of—Dr. W. G. Porter, 68.
Throat, nose and ear, Diseases of the—Dr. W. Lamb, 224.
Tongue, Cancer of the—Sir D'A. Power, 64.
Treves, Sir F.—Surgical applied anatomy, 66.
Tubercle, The early diagnosis of—Dr. C. Riviere, 61.
Vaccine treatment, Practical—Dr. R. W. Allen, 67.
Wallace, Dr. J. S.—Child welfare, 224.
Welch, W. H., Bibliography of, 224.
Wilson, Dr. R. M.—The nervous heart, 129.
Wilson, Drs. R. M. and W. M. T.—War diseases and pensions, 176.
Wingfield, Dr. H.—Forms of alcoholism, 62.
Women, Diseases of—Dr. C. Berkeley, 172.
Wood, R. C.—The soldiers' first aid, 177.
Zoology, Elementary—L. A. Borradaile, 68.

Bristol Medico-Chirurgical Society, 78, 135, 236.
Buckmaster, Dr. G. A.—Some considerations of the applications of physiology to medicine, 7.

Cerebro-spinal meningitis, Recent work on, 52.
Clarke, Dr. C.—Malaria (discuss.), 46.
Coombs, Dr. C.—Irritable heart (discuss.), 108 ; (with Drs. Stack and Rolfe)—Poisoning by "mustard" gas, 151.
Cystocele, Operation for the cure of prolapse and—Dr. W. C. Swayne, 81.

Davies, Dr. D. S.—Cases of encephalitis lethargica in Bristol, 25 ; Malaria (discuss.), 38 ; Tuberculosis and consumption in relation to public health, 211.
Davis, Dr. O. C. M.—The clinical significance of adsorption phenomena, 205.
Dawson, Lord, Visit of, 187.
Diverticulitis, 57.
Dropsy, Famine, as a food-deficiency disease—Dr. J. A. Nixon, 137.
Ductus arteriosus, Persistence of 241.
Duodenal ulcers, Medical treatment of, 124.
Duodenal ulcers, Surgical treatment of, 165.
Dyspepsia, Chronic, after "gassing," 126.

Edgeworth, Dr. F. H.—Encephalitis lethargica, 25 ; On the causes of death in lobar pneumonia, 88.
Editorial notes, 73, 130, 178, 229.
Electric stimulation of paralysed muscles, 164.
Encephalitis lethargica in Bristol—Drs. D. S. Davies, J. O. Symes, F. H. Edgeworth, I. W. Hall and J. A. Nixon, 25.
Erythræmia, Case of—Dr. G. Parker, 91.
Every-Clayton, Dr. L. E. V.—Medicine and surgery, 193.

Famine-dropsy as a food deficiency disease—Dr. J. A. Nixon, 137.

Firth, Dr. J. L.—Report on Surgery, 165.

Fortescue-Brickdale, Dr. J. M.—Report on medicine, 124.

Gas, Poisoning by mustard—Drs. E. H. E. Stack, C. Coombs, R. Rolfe, 151.

Gassing, Chronic dyspepsia after, 126.

Gastric ulcers, Medical treatment of, 124.
Gastric ulcers, Surgical treatment, 126, 165.
Gastro-enterostomy, Physiological results of, 168.
Griffiths, L. M.—Queene Elizabethes achademy, 1.

Hall, Dr. I. W.—Encephalitis lethargica in Bristol, 25.
Heart, Irritable, discussion on, 108.
Herapath, Dr. C. E. K.—Irritable heart (discuss.), 113.
Hospital amalgamation, 73, 130.
Hospital, Paying, in Bristol, 181.
Hospitals, Almoner for the voluntary, 232.
Hospitals as a public trust, The voluntary, 229.

Influenza, Tachycardia after—Dr. J. A. Birrell, 122.

Jaundice in a child, 240.

Kerfoot, Dr. S.—Malaria (discuss.), 41.

Library of the Bristol Medico-Chirurgical Society, 76, 133, 182, 234.
Local Medical Notes, 79, 136, 187, 242.
Lymphosarcoma of the tonsil and palate, Case of—Dr. P. Watson-Williams, 51.

Malaria, Discussion on, 38.
Medicine and surgery—Dr. L. E. V. Every-Clayton, 193.
Medicine, Reports on—Dr. J. M. Fortescue-Brickdale, 124; Dr. J. A. Nixon, 52; Dr. G. Parker, 162.
Meningitis, Cerebro - spinal, Recent work on, 52.
Mental conditions, Effects of the War on—R. H. Norgate, 95.
Ministry of Health, Consultative Council Report, 178, 188.
Morris, A. G.—Irritable heart, (discuss.), 119.
Mustard gas poisoning—Drs. E. H. E. Stack, C. Coombs, R. Rolfe, 151.

Nixon, Dr. J. A.—Encephalitis lethargica in Bristol, 25; Famine-dropsy as a food-deficiency disease, 137; Report on medicine, 52.
Norgate, R. H.—The effects of the war on the mental condition of the citizens of Bristol, 95.

Obituary, 184.
Omentum, Diagnosis of torsion of the great—Dr. J. Swain, 202.
Ophthalmological Society, South-Western, 242.

Palate, Case of lymphosarcoma of tonsil and—Dr. P. Watson-Williams, 51.
Paralysed muscles, Electric stimulation of, 164.
Parker, Dr. G.—A case of erythræmia or splenic polycythæmia, 91; Report on medicine, 162.
Physiology to medicine, Some considerations of the application of—Dr. G. A. Buckmaster, 7.
Pneumonia, Causes of death in lobar—Dr. F. H. Edgeworth, 88.
Prolapse and cystocele, An operation for the cure of—Dr. W. C. Swayne, 81.
Purpura hæmorrhagica, Case of, 240.

Queene Elizabethes Achademy—L. M. Griffiths, 1.

Rolfe, Drs. E. Stack, C. Coombs, and R.—Poisoning by mustard gas, 151.
Rule, Alteration of, 78.

Shingleton-Smith, L., Obituary notice of, 186.
Short, Dr. A. R.—The modern treatment of tuberculosis of the spine, 19.
Speleological Society, Bristol, 238.
Spine, Modern treatment of tuberculosis of—Dr. A. R. Short, 19.
Stack, C. Coombs and R. Rolfe, Drs. E.—Poisoning by Mustard Gas, 151.
Surgery, Reports on—Dr. J. L. Firth, 165; Dr. J. Swain, 55.
Swain, Dr. J.—Report on surgery, 55; The diagnosis of torsion of the great omentum, 202.

Swayne, Dr. W. C.—An operation for the cure of prolapse and cystocele, 81.
Symes, Dr. J. O.—Cases of encephalitis lethargica, 25.

Tachycardia, Post-influenzal—Dr. J. A. Birrell, 122.
Tonsil, Case of lymphosarcoma of the—Dr. P. Watson-Williams, 51.
Tuberculosis and consumption in relation to public health—Dr. D. S. Davies, 211.
Tuberculosis of the spine, Modern treatment of—Dr. A. R. Short, 19.
Typhoid carrier, Vesical calculus in a urinary—D. Wood, 148.

Vesical calculus in a urinary typhoid carrier—D. Wood, 148.

War on the mental conditions of citizens, The effects of the—R. H. Norgate, 95.
War œdema—Dr. J. A. Nixon, 137.
Watson-Williams, Dr. P.—Case of lymphosarcoma of the tonsil and palate, 51.
Wood, D.—Vesical calculus in a urinary typhoid carrier, 148.

